# The Effects of COVID-19 Social Restrictions and Subsequent Informal Support Limitations on Intimate Partner Violence: An Opinion Piece

**DOI:** 10.3389/fgwh.2022.829559

**Published:** 2022-06-13

**Authors:** Ryan L. Davies, Kylie Rice, Adam J. Rock

**Affiliations:** School of Psychology, University of New England, Armidale, NSW, Australia

**Keywords:** intimate partner violence, domestic violence, COVID-19, coercive control, informal support, social support network

## Introduction

Two years since the Coronavirus Disease-2019 (COVID-19) was declared a worldwide pandemic, there have been more than 460 million people infected and more than 6 million deaths (1). As the highly infectious Omicron strain continues to spread worldwide, case numbers are again trending upwards in many countries. Daily, there are on average, over a million new cases and over 5,000 deaths being recorded [as of 15/03/2022; (1)].

Similar to previous times of crisis (e.g., natural disasters), an increase in violence has been observed (2), and this has been disproportionately seen in intimate partner violence against women [IPVAW; (3, 4)]. Whilst it is noted that men can be victims of interpersonal violence, the majority of this violence is perpetrated against female intimate partners (5). Additionally, while IPVAW occurs among all social groups, ethnic minorities and migrant women are more vulnerable (6). IPVAW is categorized by acts of a physical, sexual, and/or psychological nature committed by either a current or former partner (7). IPVAW is underpinned by a perpetrator's use of coercive control, which is the patterned and repetitive use of various violent behaviors to frighten or punish the survivor (8). IPVAW can have long term negative consequences, with survivors experiencing reduced quality of life outcomes (9). This opinion article considers what risk factors COVID-19 and its restrictions have exacerbated, and how restrictions have reduced the efficacy of informal supporters.

## Increases in IPVAW

Rates of IPVAW have been observed to significantly increase during the pandemic. For example, considerable increases in IPVAW reports have been almost immediately visible when stay-at-home orders are imposed. Domestic violence helplines in both Spain and the United Kingdom reported between a 25% and 30% call increase in the first few days of lockdown protocols (10). Younger age of first experience of IPVAW has also been indicated with increased reporting from adolescent girls in Syria, Lebanon, and Ethiopia (11). In addition, it appears that IPVAW incidents are becoming more violent with police assessments in Norway showing a significant increase in the severity of violence used by perpetrators during COVID-19 lockdowns compared to the 4 years prior (12). Increased severity has also been evident during the United Kingdom's lockdown in March 2020, where domestic homicides doubled with 16 women identified as being murdered by their partner (13).

## COVID-19 Restrictions and IPV: More Risk, Less Help

### COVID-19 Related Risk Factors

To slow the spread of COVID-19, various measures to contain its transmission have been implemented. Given the nature of transmission these measures have largely targeted social interactions, with social distancing and stay-at-home orders widely utilized. Whilst social restrictions have been necessary, there has been unintended consequences of escalating risk of IPVAW (10). COVID-19 and the associated restrictions have resulted in an escalation of three key socially related risk factors. Namely, restrictions have resulted in survivors being socially isolated, having greater dependence on the perpetrator, and experiencing poorer mental health (14–16).

### Decreases in Efficacy and Availability of Informal Support

In addition to exacerbating risk factors, COVID-19 restrictions have significantly reduced the protective capacity of social networks to mitigate the risk of violence and keep survivors safe (17). Informal supporters, such as family members, friends, colleagues, and neighbors are an important source of support for most survivors experiencing IPVAW (18). Many survivors' first step toward help is to disclose the occurrence of abuse to informal supporters (19) or have informal supporters recognize the signs of abuse (20). Typically, informal supporters provide emotional, instrumental, and informational support, which ultimately reduces the risk experienced by the survivor (21). In their qualitative study, Gregory and Williams (15) identified that due to the myriad of social restrictions informal supporters found it difficult to accurately assess the risk level of IPVAW and provide appropriate responses.

### Poor Psychological Health

The consequences of IPVAW can have ongoing profound psychological impacts for a survivor. It has been widely found that survivors experience depression, posttraumatic stress disorder, and suicidal ideation at three to five times the rate of women who have not experienced IPVAW (22–24). Poor psychological health can reduce the likelihood of a survivor escaping IPVAW and make them more vulnerable to repeated victimization (25). In a global study during the pandemic, rates of depression have been rising with greater increases observed in women than men (26). Emerging evidence also indicates that while women are more likely to experience poor mental health during the pandemic, the severity of these symptoms is greater in women who are experiencing IPVAW (4). For example, in a study of Ugandan women, Miller et al. (16) found that women who had experienced IPVAW during the pandemic had significantly higher scores on measures of depression and COVID-19 anxiety.

However, most survivors do not engage with formal mental health services, instead seeking emotional support from informal supporters (18). Survivors who have maintained strong social support networks throughout the pandemic have been found to have a significantly reduced risk of developing depressive symptoms then their counterparts with weaker social networks (27). However, COVID-19 restrictions limit the ability for informal support networks to provide emotional support (e.g., validation of emotions, and encouragement) to survivors creating greater vulnerability for adverse mental health outcomes and IPVAW risk.

### Social Interruption / Isolation

An objective of a perpetrator's use of coercive control is to isolate the survivor thereby increasing their control of the survivor and minimizing opportunity for the violence to be detected. Controlling tactics (e.g., threats and stalking) can result in the survivor having limited contact with their social support network (28). Reductions in social interaction result in fewer opportunities for perpetrators to be held accountable for their actions and can, therefore, exacerbate the risk of IPVAW (29). COVID-19 restrictions and public health recommendations to minimize social interactions can be exploited by perpetrators to isolate survivors in order to increase the level of control held (15).

Informal support networks are a key source of informational support for survivors. Informational support can include advice, guidance, and provision of useful information to the survivor which complements the emotional support fostered through strong social networks (30). Furthermore, as informal supporters are typically well placed to identify IPVAW they can be a safe link for survivors to engage with formal domestic violence support services (31). However, many COVID-19 restrictions have reduced the opportunity for survivors to have meaningful social engagement with informal supporters where a disclosure could be made (17). For example, the cancellation of organized activities has resulted in many survivors not having contact with key social supports. Additionally, stay-at-home orders create complete social isolation and make informal supporters incapable of identifying IPVAW (32). Furthermore, even when opportunities for social engagement are present, social distancing requirements have resulted in diminished quality of interactions (33). For example, incidental exchanges are less frequent, often much briefer, and may be restricted to surface level topics, thus not lending themselves to sensitive conversations. With limited social opportunity language, barriers can also strain the building and maintenance of social networks as has been observed in immigrant populations (34). Additionally, wearing a face mask can result in critical non-verbal cues being ambiguous and subject to misinterpretation (33, 35), while also potentially hiding physical signs of abuse (e.g., bruising, cuts, abrasions). Finally, there has been a strong shift to use electronic communications for social interactions which also reduces visual information and depth of discussion (36). This myriad of changes impacting interpersonal interactions reduce the opportunity for an informal supporter to identify that help is required.

### Perpetrator Dependence

Perpetrators of IPVAW use controlling tactics to reduce the level of independence of a survivor (37). For example, financial independence increases the likelihood that a survivor will escape an abusive relationship and, therefore, perpetrators will use tactics to limit a survivor's engagement in employment and access to financial resources (38). Ultimately, the consequence of coercive tactics results in global reductions of independence and increased dependence on the perpetrator. COVID-19 has reduced survivors' independence with higher levels of unemployment and financial stress (14). By the end of July 2020, almost ten million Americans had become unemployed because of the pandemic, placing considerable strain on millions of households (14). While a significant portion of these people have since been able to return to work, young women have seen significantly reduced levels of re-employment than their male counterparts (39). Impacts of economic insecurity and subsequent food insecurity have been widely felt in rural Bangladesh, where 55% of female participants identified experiencing IPVAW because of loss of income attributed to lengthy lockdowns (40). Furthermore, women with a disability experience greater vulnerability given that in addition to financial dependence they can also experience physical dependence on the perpetrator (41). For vulnerable women experiencing IPVAW, unemployment can increase the risk of ongoing victimization by contributing to household financial stress, blame from the perpetrator, a reduction in the survivor's visibility in the community, and reduced financial independence should a woman decide to leave an abusive partner (38). Ultimately, this lack of independence leaves the survivor reliant on the perpetrator.

Typically, in situations of economic insecurity social networks can provide instrumental support to survivors. This can be though provision of financial assistance or refuge and linking the survivor to relevant formal support services (42). However, given social restrictions the identification of IPVAW and abilities of an informal supporter to safely engage with a survivor are limited. Gregory and Williams (15) found that often informal supporters were also experiencing financial strain, and this coupled with their own additional care responsibilities (e.g., home-schooling children) meant that their capacity to help survivors was greatly reduced.

## The Triangle of Social Risk Factors

**Figure 1:**
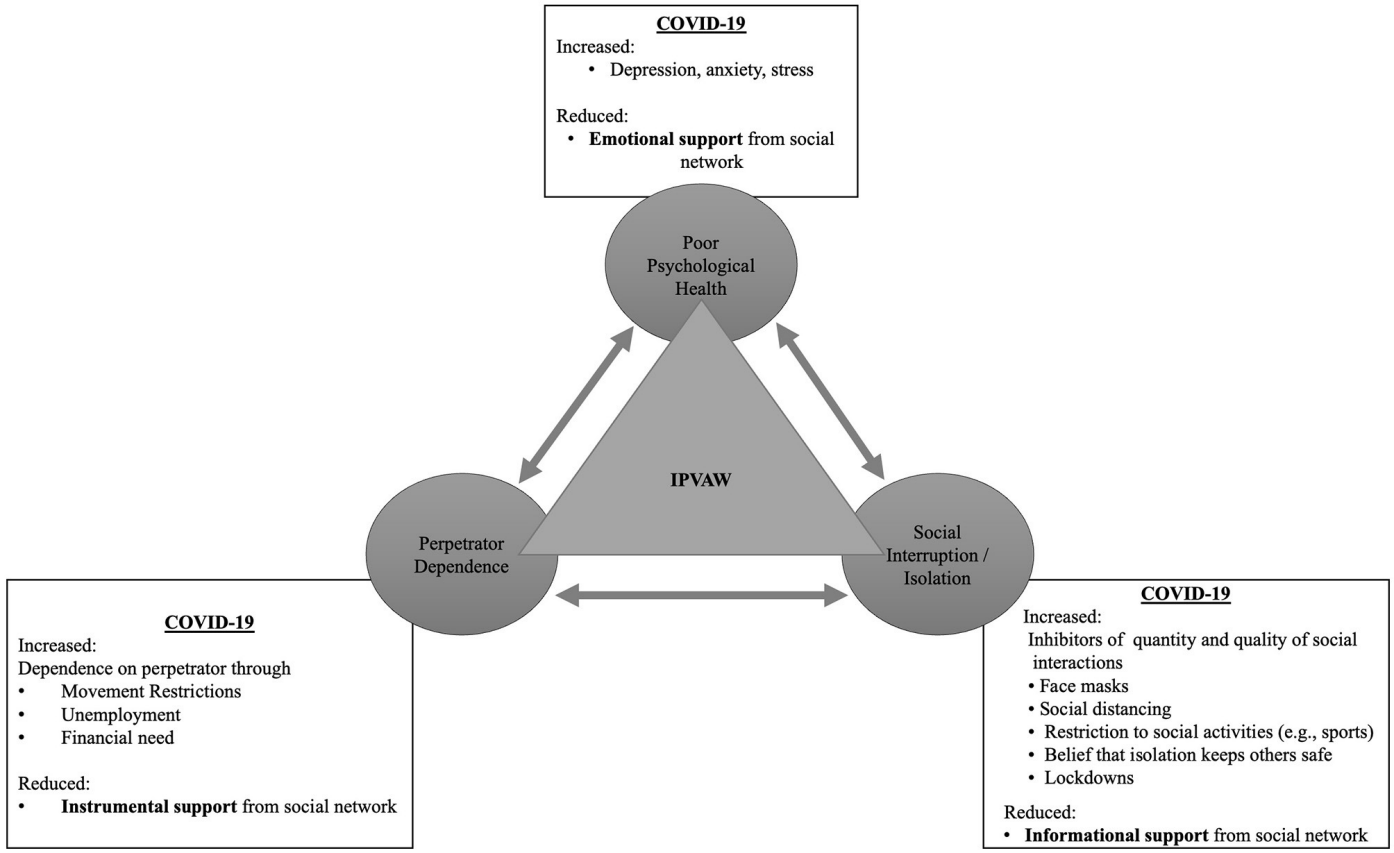
COVID-19 social restriction as additional stressors to IPV risk factors.

It is clear that the restrictions imposed to address the spread of COVID-19 has had a deleterious impact on IPVAW. While there are numerous risk factors of IPVAW, the three socially driven risk factors of poor psychological health, social isolation / interruption, and perpetrator dependence have been independently found to increase risk of IPVAW during the pandemic (14–16). However, it is proposed by the present authors that these three social factors are interconnected and together influence the risk of IPVAW. Figure 1 demonstrates the relationship between the social risk factors as well as summarizing the exacerbating impact of COVID-19 restrictions and the inhibiting impact on the efficacy of informal supporters.

## Conclusion

The COVID-19 pandemic has seen a significant increase in social risk factors of IPVAW and subsequent escalations in the frequency and severity of violence (12). Given the longevity of the pandemic, there is considerable concern regarding the lasting consequences of stringent COVID-19 restrictions. It is likely that for some time social circles will remain smaller with survivors having infrequent meaningful contact with others (43). Informal supporters play a vital role in the safety and wellbeing of survivors of IPVAW, and this includes supporting survivors to engage with specialist supports. However, longstanding social restrictions resulting from COVID-19 have impacted the abilities of informal supporters and therefore reduced opportunity for survivors to engage with specialist services. Therefore, it is imperative that health professionals routinely screen for IPVAW with their patients to improve detection rates and have appropriate referral pathways for patients experiencing violence.

Finally, the reduced efficacy of informal supporters suggests that survivors may be exposed to violence for longer periods without intervention. The combination of continued IPVAW and compromised informal support could have potentially fatal outcomes for many women. It is therefore imperative that governments and domestic violence services have appropriate strategies to minimize the impact of socially related risk factors, such as streamlining financial aid for survivors in order to remove financial dependence on a perpetrator, and are adequately resourced to address this burgeoning social crisis.

## Author Contributions

RD conceived of the presented idea. KR and AR provided constructive feedback and helped shape the narrative presented. All authors contributed to the article and approved the submitted version.

## Conflict of Interest

The authors declare that the research was conducted in the absence of any commercial or financial relationships that could be construed as a potential conflict of interest.

## Publisher's Note

All claims expressed in this article are solely those of the authors and do not necessarily represent those of their affiliated organizations, or those of the publisher, the editors and the reviewers. Any product that may be evaluated in this article, or claim that may be made by its manufacturer, is not guaranteed or endorsed by the publisher.
